# Editorial on the Special Issue: Regulation by Non-Coding RNAs

**DOI:** 10.3390/ijms141121960

**Published:** 2013-11-06

**Authors:** Nicholas Delihas

**Affiliations:** Department of Molecular Genetics and Microbiology, School of Medicine, Stony Brook University, Stony Brook, NY 11794-5222 USA; E-Mail: nicholas.delihas@stonybrook.edu; Tel.: +1-631-286-9427; Fax: +1-631-632-9797

**Keywords:** non-coding RNAs (ncRNAs), microRNAs (miRNAs), long ncRNAs (lncRNAs), Alu sequences, ncRNAs and human disease processes, regulation of ncRNA expression, ncRNAs and cell differentiaton

## Abstract

This Special Issue of *IJMS* is devoted to regulation by non-coding RNAs and contains both original research and review articles. An attempt is made to provide an up-to-date analysis of this very fast moving field and cover regulatory roles of both microRNAs and long non-coding RNAs. Multifaceted functions of these RNAs in normal cellular processes, as well as in disease progression, are highlighted.

These are exciting times for RNA molecular biologists, especially those concentrating on eukaryotic non-coding RNAs (ncRNAs)! New RNAs are constantly being found, e.g., thousands of large circular RNAs have recently been identified in human cells, some of which are shown to act as regulators that bind and sequester microRNAs [1]. The continuous discovery of new RNAs provides a rich environment for future RNA research and affords the possibility of many new functions being discovered.

This Special Issue of *IJMS* [2] contains both original research and review articles, and is devoted to regulation by ncRNAs. Multifaceted roles of these RNAs in normal cellular functions, as well as in disease processes, are highlighted. To mention a few, long ncRNAs (lncRNAs) and microRNAs (miRNAs) are both intimately involved in hematopoietic differentiation [3]. ncRNAs play a role in the adaptive immune response [4] and in diseases such as muscular dystrophies [5] and cancers [6–14]. Another paper discusses roles of lncRNAs in the pathogenesis of haematological malignancies [15]. The budding field of miRNAs and possible lncRNAs functions in cardiovascular disease is presented [16], as well as miRNA functions in abdominal aortic aneurysm [17]. In addition, it was shown that miRNAs regulate the expression of the Huntingtin gene *HTT* [18]; mutations in *HTT* cause Huntington’s disease. There is also a review on the 3′ non-coding region of mRNAs, which discusses alterations in 3′ UTRs sequences of mRNAs that may contribute to the development of various diseases in humans [19]. The fascinating viral immune system in prokaryotes, the RNA-based CRISPR complex is presented [20], and on “the other-side of the coin”, the intricate mechanism of evasion of host RNA-directed DNA methylation of single-stranded DNA viruses by the land plants [21]. The intimate genomic association of transposable elements and their remnants with ncRNA genes is also presented, and how lncRNA Alu-containing transcripts can participate in disease formation when mutated, such as the formation of brainstem cell atrophy that leads to death [22]. Highlighted also are the Alu-lncRNAs/Alu-mRNA interactions that inhibit mRNA expression. In addition, one paper is devoted to computational methods in comparative genomics in light of the availability of new sequencing technologies [23]. Another paper surveys the current knowledge on regulation of miRNAs, principles of target recognition, and highlights new and novel non-canonical functions [24], whereas a separate paper also discusses techniques for the prediction of microRNA targets [25]. However this Issue includes numerous other highly informative papers describing the interesting roles of miRNAs and lncRNAs in various biological processes and the regulation of these ncRNAs [26–46]. In all, there are 44 papers, which attest to the large amount of interest and research activity in ncRNA molecular biology.

## Conclusions

We indeed hope the readers will enjoy this Special Issue of *IJMS* and an attempt to present up-to-date findings in the rapidly moving and exciting field of non-coding RNA molecular biology and involvement of RNA in disease development.
